# Promoting mental health in esports

**DOI:** 10.3389/fpsyg.2024.1342220

**Published:** 2024-01-19

**Authors:** Jolan Kegelaers, Michael G. Trotter, Matthew Watson, Ismael Pedraza-Ramirez, Iván Bonilla, Paul Wylleman, Olivier Mairesse, Martijn Van Heel

**Affiliations:** ^1^Vrije Universiteit Brussel, Brussels, Belgium; ^2^Umeå University, Umeå, Sweden; ^3^German Sport University Cologne, Cologne, Germany; ^4^G2 Esports, Berlin, Germany; ^5^Universitat Autònoma de Barcelona, Barcelona, Spain

**Keywords:** anxiety, depression, esports, gaming, stressors, well-being, e’athletes

## Abstract

There is growing recognition of the demands and health challenges experienced by esports athletes. The purpose of this perspective paper is to draw specific attention to the mental health of competitive gamers and spur on both future research and applied initiatives focussing on this important but under-addressed topic. We will briefly discuss the prevalence of mental health concerns, domain-specific stressors, and the need for comprehensive mental health support systems tailored to the esports context. It is our hope that, with this perspective paper, we can help set a new research agenda addressing mental health in esports.

## Background

Esports forms one of the fastest-growing global entertainment industries. In 2022 alone, an estimated 921 million viewers worldwide watched esports content, with an associated annual revenue of up to 1.4 billion dollars.^1^ At the policy level, the value and benefits of esports are widely acknowledged. To illustrate, a recent report commissioned by the European Parliament highlighted that esports can form an important platform for the promotion of positive prosocial values, such as fair play and teamwork, and the development of crucial skills for a digital society (Scholz and Nothelfer, 2022). Nevertheless, the rapid growth of the industry equally poses several novel challenges. *E’athletes*, defined as “individuals who compete in any esports to achieve an in-game ranking or who compete in a formalized competition” (Bubna et al., 2023, p. 3), may face a number of domain-specific demands and health challenges (Monteiro Pereira et al., 2022; Schary et al., 2022). The purpose of this perspective paper is to draw specific attention to the mental health of e’athletes. The relatively young age of most e’athletes (i.e., teens to early twenties) corresponds to the typical age of onset for many common mental disorders (Kessler et al., 2007). This fact combined with the unique stressors experienced within the esports environment may pose a distinct risk for their mental health. Nevertheless, attention for mental health in esports remains scarce. As such, the aim of this paper is to encourage future research as well as evidence-based applied initiatives focussing on this important but under-addressed topic.

## Mental health concerns in e’athletes

Research on mental health in esports remains in its infancy. Moreover, discussions often remain limited to gaming addiction (Kuss and Griffiths, 2012; Schary et al., 2022), which reflects only a small part of the broad range of potential mental health concerns faced by e’athletes. One of the first studies to study a range of common mental disorders found that around 37% of a sample of Portuguese electronic football players experienced symptoms of depression and anxiety, whereas 45% experienced symptoms of sleep disturbance (Monteiro Pereira et al., 2021). Another small-scale study with Australian, US, and Korean e’athletes equally found relatively high symptoms of depression (although no exact prevalence rates were reported), which were strongly correlated with sleep disturbance and total training hours (Lee et al., 2021). These important first studies highlight that mental health concerns are rather common in esports and may be on par with prevalence rates seen in traditional sports (Gouttebarge et al., 2019). Nevertheless, these studies are also limited by their relatively small sample sizes, as well as the limited range of included esports titles. As such, there remains a clear need for more high-quality research on the prevalence of common mental disorders across a range of different game titles. Whenever possible, such future research should rely on diagnostic clinical interviews, in addition to self-report instruments, to obtain accurate prevalence rates of common mental disorders (Lundqvist and Andersson, 2021).

At the same time, it should be acknowledged that mental health entails more than the absence of mental disorders (World Health Organization, 2018). Keyes (2002) suggests that mental ill-health (i.e., prevalence of mental disorders) and positive mental health reflect two related but essentially distinct continua. Such positive mental health is typically considered under the rubric of emotional, psychological, or social well-being (Keyes, 2002). To the best of our knowledge, only a single study has examined the well-being of competitive gamers. Kocadağ (2019) found that both the desire to pursue an esports career as well as total time practicing esports were negatively associated with psychological well-being in young people. Building on this work, researchers should consider indicators and determinants of more positive states of functioning and well-being to gain insight into the “complete state” of e’athletes’ mental health. Importantly, recognizing that research in general psychology has often been limited by considering well-being as a vague “feel-good factor” (Lundqvist and Andersson, 2021), future research should adopt theoretically-informed and empirically robust measures to assess well-being in esports.

## Stressors in esports

When discussing mental health, it is also important to examine the unique stressors and demands associated with esports and their impact on players’ health and well-being. Research in traditional sports has demonstrated that domain-specific stressors can have a profound impact on athletes’ mental health (Kuettel and Larsen, 2020; Kegelaers et al., 2022). Similarly, recent studies have started to elucidate some of the unique stressors faced by e’athletes (Smith et al., 2019; Leis and Lautenbach, 2020; Poulus et al., 2022). For example, competitive gamers often face performance demands in the form of dealing with performance pressure and expectations, managing in-game performances, and coping with defeat. At the team level, players may face stressors related to communication difficulties, interpersonal conflicts, and antisocial or “toxic” team behaviors. These challenges may be further compounded by the low levels of received social support reported by e’athletes (Trotter et al., 2021). At the organizational level, players may be confronted with increasing public scrutiny and criticism from audiences and media, as well as unprofessional organizational structures and support. Female e’athletes may also be confronted with increased stigma, gender discrimination, and social barriers related to hegemonic masculinity, expected gender roles, or online harassment (Scholz and Nothelfer, 2022).

E’athletes may also face personal stressors relating to unhealthy lifestyles, work-life imbalance, and excessively long practice times. For example, a qualitative study on practice designs in League of Legends (LoL) players found that a culture of “grinding” and overtraining placed a physical and emotional toll on players and could negatively impact their well-being (Abbott et al., 2022). The sedentary nature of professional gaming, involving prolonged sitting and repetitive movements, has also been linked to musculoskeletal problems, including chronic lower back pain (Lam et al., 2022), which has in turn been associated with anxiety and depression symptoms, as well as insomnia complaints, especially within a young demographic (Bilterys et al., 2021). Sleep disturbances may be further compounded by offset sleep patterns and late night screen exposure (Goulart et al., 2023; Santos et al., 2023). Consequently, unhealthy lifestyle habits, intensified by gaming culture and dysfunctional beliefs about sleep, may lead to poor sleeping habits, sleep disturbance, and associated mental health concerns in e’athletes (Bonnar et al., 2019).

Despite growing awareness of some of the typical stressors and demands associated with esports, little is known about their impact on e’athletes’ mental health. To date, a single study has focused on the direct impact of esports-related stressors, demonstrating that stressors such as game uncertainty, personal concerns, in-game pressure, and sleep quality significantly predict symptoms of depression and anxiety in players (Smith et al., 2022). Building on this work, more research is needed to establish the domain-specific risk factors which impact e’athletes’ mental health as well as how such stressors may differ depending on player status or game title. Additionally, future research should consider a developmental perspective to study how specific esports-related stressors or demands can differ or intensify depending on specific career stages or transitions (Wylleman et al., 2015).

## Mental health support systems in esports

Given the potential risk for mental health concerns, there is also a clear need for evidence-based mental health support frameworks in esports. Duty of care for e’athletes’ mental health should be shared by all stakeholders within the esports landscape and necessitates a structured support system (Hong, 2022). Although comprehensive mental health support systems remain absent within esports (Schary et al., 2022), inspiration may be drawn from recent developments in traditional sports (e.g., Purcell et al., 2019; Kegelaers et al., 2023). In general, such support systems should focus on the development of e’athletes’ knowledge and self-management competencies which help them manage experienced stressors, promote or maintain their well-being, and prevent the onset of mental health problems. For example, early intervention focused on health education and sleep intervention is recommended to counteract the impact of gaming culture on maladaptive sleep and daytime habits (Bonnar et al., 2019). Coping effectiveness training programs tailored to the esports context may help e’athletes manage their experienced demands and strengthen their resilience (Poulus et al., 2023). Finally, mental health literacy interventions may focus on increasing awareness of mental health in e’athletes (Breslin et al., 2022). Mental health literacy is defined as “understanding how to obtain and maintain positive mental health; understanding mental disorders and their treatments; decreasing stigma related to mental disorders; and, enhancing help-seeking efficacy” (Kutcher et al., 2016, p. 155). Evidence from traditional sports suggests that such awareness-building interventions are effective at decreasing stigma and improving mental health symptom recognition, help-seeking intentions and behavior, confidence to support others, and overall well-being (Breslin et al., 2022).

Although developing self-management competencies forms a crucial part of any structured support system, solely focussing on such interventions risks neglecting the influence of the broader environment (Purcell et al., 2019). As such, key stakeholders (e.g., coaches, parents, performance staff, tournament/league organizers, game developers) within the esports landscape equally need to be equipped to promote mental health and to recognize and adequately respond to potential mental health concerns. For example, research from traditional sports suggests that mental health literacy interventions may not only be beneficial for individual athletes, but should also be targeted at the broader entourage around the athlete to aid them in recognizing early warning signs and improve referral efficacy (Breslin et al., 2022). Recognizing the role of key stakeholders, and in particular coaches, is crucial since there currently exists a lack of codified career development pathways or formal coach education initiatives in esports (Watson et al., 2022). Moreover, coach practices in esports are currently primarily focused on short-term performances, often neglecting the long-term holistic development and well-being of e’athletes (Watson et al., 2022). As such, there is need to raise industry-wide awareness regarding the role of key stakeholders, as well as offering formal educational pathways to upskill them in the area of mental health.

Finally, within any structured mental health support system there is a need to ensure access to specialist multi-disciplinary mental health care in case of severe mental health concerns or clinical disorders (Purcell et al., 2019; Kegelaers et al., 2023). To ensure effective and timely access to appropriate mental health care, esports organizations need to consider the formal or informal ways through which early indicators of mental health problems can be detected, signaled, and acted upon. As stated, coaches and other stakeholders may play a crucial role in informally detecting potential warning signs and guiding e’athletes toward available help resources. Additionally, organizations may consider more formal approaches to mental health screening at key points during the competitive season (Purcell et al., 2019). Ensuring access to specialized mental health care also requires established referral structures to in-house or out-house specialized and accredited mental health professionals (e.g., clinical or counseling psychologists, psychiatrists). Ideally, such experts possess context-specific knowledge and understanding of how esports-related characteristics and demands can impact e’athletes’ mental health. This includes adapting traditional interventions to e’athletes’ unique lifestyle by managing game-related effects, accommodating unconventional training times, and implementing therapeutic approaches tailored to esports. As such, there is an urgent need for educational programs which allow mental health professionals to specialize in esports.

## Conclusion

In summary, the rapid growth of the esports industry poses a challenge to the mental health of a growing number of individual e’athletes. Organizations hold a crucial responsibility for safeguarding and promoting the mental health of their employees. Nevertheless, both research and applied work in this domain remain in its infancy. The current perspective paper hopes to contribute to a novel research and policy agenda addressing this important topic in esports.

## Data availability statement

The original contributions presented in the study are included in the article/supplementary material, further inquiries can be directed to the corresponding author.

## Author contributions

JK: Conceptualization, Writing – original draft, Writing – review & editing. MT: Conceptualization, Writing – review & editing. MW: Writing – review & editing. IP-R: Writing – review & editing. IB: Writing – review & editing. PW: Writing – review & editing. OM: Writing – review & editing. MV: Conceptualization, Writing – review & editing.

## Footnotes

^1^Data available from Newzoo 2022 Global Esports and Live Streaming Market Report: https://newzoo.com/resources/trend-reports/newzoo-global-esports-live-streaming-market-report-2022-free-version.
